# Genetic analysis of the spindle checkpoint genes *san-1*, *mdf-2*, *bub-3 *and the CENP-F homologues *hcp-1 *and *hcp-2 *in *Caenorhabditis elegans*

**DOI:** 10.1186/1747-1028-3-6

**Published:** 2008-02-04

**Authors:** Vinita A Hajeri, Anil M Stewart, Landon L Moore, Pamela A Padilla

**Affiliations:** 1 Department of Biological Sciences, University of North Texas, Denton, TX, USA; 2 Department of Genetics and Genomics, Boston University School of Medicine, Boston, MA, USA

## Abstract

**Background:**

The spindle checkpoint delays the onset of anaphase until all sister chromatids are aligned properly at the metaphase plate. To investigate the role *san-1*, the MAD3 homologue, has in *Caenorhabditis elegans *embryos we used RNA interference (RNAi) to identify genes synthetic lethal with the viable *san-1(ok1580) *deletion mutant.

**Results:**

The *san-1(ok1580) *animal has low penetrating phenotypes including an increased incidence of males, larvae arrest, slow growth, protruding vulva, and defects in vulva morphogenesis. We found that the viability of *san-1(ok1580) *embryos is significantly reduced when HCP-1 (CENP-F homologue), MDF-1 (MAD-1 homologue), MDF-2 (MAD-2 homologue) or BUB-3 (predicted BUB-3 homologue) are reduced by RNAi. Interestingly, the viability of *san-1(ok1580) *embryos is not significantly reduced when the paralog of HCP-1, HCP-2, is reduced. The phenotype of *san-1(ok1580);hcp-1(RNAi) *embryos includes embryonic and larval lethality, abnormal organ development, and an increase in abnormal chromosome segregation (aberrant mitotic nuclei, anaphase bridging). Several of the *san-1(ok1580);hcp-1(RNAi) *animals displayed abnormal kinetochore (detected by MPM-2) and microtubule structure. The survival of *mdf-2(RNAi);hcp-1(RNAi) *embryos but not *bub-3(RNAi);hcp-1(RNAi) *embryos was also compromised. Finally, we found that *san-1(ok1580) *and *bub-3(RNAi)*, but not *hcp-1(RNAi) *embryos, were sensitive to anoxia, suggesting that like SAN-1, BUB-3 has a functional role as a spindle checkpoint protein.

**Conclusion:**

Together, these data suggest that in the *C. elegans *embryo, HCP-1 interacts with a subset of the spindle checkpoint pathway. Furthermore, the fact that *san-1(ok1580);hcp-1(RNAi) *animals had a severe viability defect whereas in the *san-1(ok1580);hcp-2(RNAi) *and *san-1(ok1580);hcp-2(ok1757) *animals the viability defect was not as severe suggesting that *hcp-1 *and *hcp-2 *are not completely redundant.

## Background

The spindle checkpoint genes regulate chromosome segregation during mitosis and when mutated lead to a variety of human health concerns including cancer [1,2]. Thus, it is of interest to determine genetic interactions with spindle checkpoint genes. The spindle checkpoint genes, originally identified in yeast, include Mad1, Mad2, BubR1/Mad3, Bub1, Bub2, Bub3, and Mps1 [3]. With the exception of Bub2, all of these genes are conserved between yeast and vertebrates. In vertebrate cells there are additional gene products involved in spindle checkpoint activity indicating that there are some mechanistic differences in metazoan and fungi spindle checkpoint activity. For example, CENP-E, a kinesin motor protein that binds BubR1, is required for checkpoint establishment and, ZW10 and ROD are required for checkpoint signaling in vertebrates cells [4].

The spindle-checkpoint signaling pathway is well understood and has been discussed in many reviews [1,5-9]. Briefly, the spindle checkpoint gene products function at the kinetochore to inhibit the onset of anaphase until chromatids are properly attached to spindle microtubules. The spindle checkpoint is signaled by unattached kinetochores or a reduction in spindle microtubule tension [10-12]. In cell culture Mad1 and Mad2 localize to unattached kinetochores but not to attached kinetochores that lack tension. Bub1 and BubR1/Mad3 localize to kinetochores that either lack tension or microtubule attachment. The downstream target of the spindle checkpoint is the multi-protein E3 ubiquitin ligase, anaphase-promoting complex/cyclosome (APC/C) [13]. A complex consisting of Mad2, Cdc20, Mad3/BubR1 and Bub3 is an inhibitor of APC/C [14-16]. It is thought that Mad-2 and BubR1/Mad3 act synergistically to inhibit APC/C activity. Inactivation of the spindle checkpoint releases APC/C and cyclin B, which are required for the onset of anaphase [17]. The APC/C ubiquinates proteins, such as securin, and targets them for protein degradation. The destruction of securin leads to the release of separase and the destruction of cohesin resulting in a disruption of sister chromatid cohesion.

Much of the work to elucidate the mechanistic function of the spindle checkpoint proteins has been conducted in yeast and vertebrate cell culture. However, there is increasing evidence that the spindle checkpoint is also required for specific functions in *Caenorhabditis elegans *and *Drosophila melanogastor*. For example, in *C. elegans *the genes *mdf-1 *and *mdf-2 *(MAD1 and MAD2 homologues respectively) have a role in development and germline function [18]. Specifically, embryonic lethality, gonad defects, and a reduced brood size were observed in *mdf-1(gk2) *animals. A genetic screen for suppressors of the weak allele of *mat-3*, an APC subunit, identified several *mdf-2*,*mdf-1*, and *san-1/mdf-3 *alleles, providing evidence that the metaphase to anaphase transition in meiosis is regulated by the spindle checkpoint [19]. The spindle checkpoint is also thought to function in *C. elegans *embryonic blastomeres [20]. Briefly, the *mdf-2 *and *san-1 *(MAD3 homologue) genes are required for response to microtubule-depolymerizing environments such as anoxia-induced suspended animation and nocodazole [21,22]. Finally, the MDF-2 and SAN-1 proteins localize to the kinetochore, which is consistent with the localization of spindle checkpoint proteins in other systems [18,21]. Together, these studies indicate that the spindle checkpoint gene products function in developing organisms and are required for response to and survival of environments that alter microtubule structure.

In *C. elegans *embryos HCP-1, the CENP-F like protein, is localized to the kinetochore during mitosis; yet *hcp-1(RNAi) *animals do not have any obvious phenotype [23]. HCP-1 and HCP-2 share 42% identity (54% similarity) to one another, and HCP-1 is 29% identical (45% similar) to the CENP-F repeats [23,24]. However it is not clear if both HCP-1 and HCP-2 are orthologues of CENP-F and if HCP-1 and HCP-2 are completely redundant. Co-depletion of HCP-1 and HCP-2 by RNA interference (RNAi) results in abnormal chromosome segregation and embryonic lethality indicating a functional role in chromosome segregation [23,25]. The HCP-1/2 proteins function in kinetochore assembly. Localization of HCP-1/2 requires the kinetochore proteins HCP-3 (CENP-A), HCP-4 (CENP-C), KNL-1 and BUB-1 [23,25,26]; and HCP-1 and HCP-2 are required for the proper localization of the microtubule and kinetochore associated protein CLASP/CLS-2 [27]. Additionally, HCP-1 and HCP-2 are required for spindle checkpoint function, however it is not clear whether this requirement is direct or indirect [20].

In this report we further investigate the functional role of the spindle checkpoint gene *san-1 *by using RNAi to identify genes that are synthetic lethal with *san-1(ok1580)*. The viability of *san-1(ok1580) *embryos is significantly reduced when HCP-1, MDF-2 (MAD-2 homologue) or BUB-3 (gene Y54G9A.6, predicted to encode BUB-3 homologue) are reduced by RNAi. The viability of *mdf-2(RNAi);hcp-1(RNAi) *embryos but not *bub-3(RNAi);hcp-1(RNAi) *embryos was compromised suggesting that HCP-1 interacts with a subset (*san-1 *and *mdf-2*) of the spindle checkpoint components. Phenotype analysis of *san-1(ok1580);hcp-1(RNAi) *embryos supports the idea that HCP-1 functions in the proper alignment of chromosomes and chromosome segregation and that these functions are dependent upon the spindle checkpoint pathway.

## Results

### Phenotype analysis of *san-1(ok1850)*

Previously, it was shown that *san-1(RNAi) *embryos are viable, yet sensitive to conditions that depolymerize microtubules (anoxia or nocodazole treatment), providing evidence that the *san-1 *gene product has a role in the spindle checkpoint pathway [21,22]. To investigate the role *san-1 *has in *C. elegans *we obtained a *san-1 *deletion strain from the *C. elegans *Knockout Consortium. We verified this deletion by DNA sequence analysis and found that the *san-1(ok1580) *allele has a 992 kb deletion, which is consistent with what is documented in wormbase. The *san-1 *gene contains 8 predicted exons, of which the deletion in *san-1(ok1580) *removes all of exons 3–7 and the first 13 bp of intron 7. Thus, the *san-1(ok1580) *allele is minimally a catalytic null as the predicted kinase domain and Mad3/Bub1 homology region predicted to be essential for binding to CDC20p is absent (data not shown).

**Figure 1:**
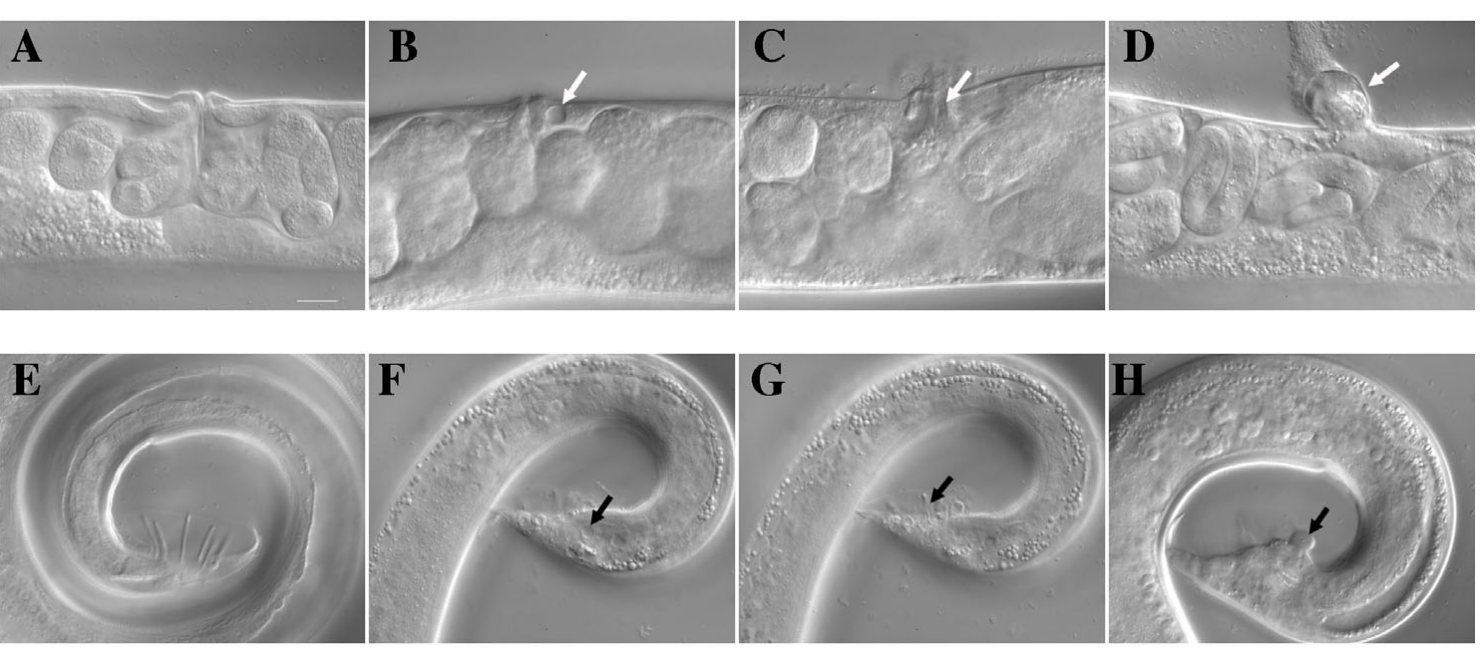
**Abnormal vulva and male tail development in the *san-1(ok1580) *animal**. L1 larvae were grown at 20°C for three days and 1-day old adults were analyzed using DIC microscopy. Wild-type hermaphrodites (A), *san-1(ok1580) *hermaphrodites (B-D), wild-type males (E) and *san-1(ok1580) *males (F-H) were analyzed for morphologic defects. White arrows point to vulva defects including tissue damage (B), abnormal morphology (C), and protruding vulva (D). Black arrows point to defects observed in the male tail, including tissue damage (F), ray and fan defects (G), and abnormal tissue structure (H). Bar = 25 μm.

Others have shown that *san-1(ok1580) *hermaphrodites grown at 24°C have several phenotypes including a higher incidence of males in the population, egg laying defects, and a higher death rate in adult hermaphrodites due to vulva bursting or formation of a bag of worms [19]. As prior work with *san-1(RNAi) *was done at 20°C, we characterized the phenotype of *san-1(ok1580) *animals grown at 20°C. Similar to animals maintained at 24°C, we found that the *san-1(ok1580) *embryos were viable, yet had some low penetrant phenotypes (Table 1). In comparison to wild-type animals, the *san-1(ok1580) *animals have a larvae arrest and slow growth phenotype (Table 1). The *san-1(ok1580) *adults have a higher incidence of death resulting from defects in vulva morphogenesis (Table 1). We further investigated these vulva defects using DIC microscopy to examine the vulva region of *san-1(ok1580) *animals and determined that the *san-1(ok1580) *animals contained tissue damage, abnormal vulva anatomy, and an increase in the protruding vulva phenotype (Figure 1B–D, Table 1). In comparison to wild-type adults, the average number of eggs held within the uterus of *san-1(ok1580) *animals is not significantly different (P = .176) (Table 1). However, the number of eggs held in the uterus was significantly different, ranging from 8 to 18 for wild-type animals, and 5 to 27 for *san-1(ok1580) *animals, indicating that some of the *san-1(ok1580) *animals were not laying embryos. Consistent with this idea, we observed that 18% (n = 16) of the *san-1(ok1580) *1-day old adults had larvae hatching out in the uterus. We analyzed progeny production in *san-1(ok1580) *hermaphrodites and determined that the average number of progeny produced was significantly decreased in comparison to wild-type hermaphrodites (Table 1). Some of the *san-1(ok1580) *hermaphrodites died as young adults and produced less than 60 progeny, whereas others did not die, yet they still produced significantly less progeny than wild-type hermaphrodites (P = .001). The *san-1(ok1580) *animals also have a higher incidence of males (Table 1). Additionally, *san-1(ok1580) *males have various defects in tail morphology including an increase in tissue damage, and abnormal ray morphology or tissue structure (Figure 1F–H). In our studies we used the *san-1(ok1580) *males for mating indicating that the *san-1(ok1580) *males are not sterile. In summary, these results are consistent with defects in cell cycle progression leading to abnormal vulva development in the hermaphrodite and somatic cell defects in the adult male [28].

**Table 1:** Phenotype analysis of *san-1(ok1580) *hermaphrodites

Phenotype	**Wild-type**	***san-1(ok1580)***
Larval arrest (%)	0.0	2.8 ± 1.5
Slow growth (%)	0.0	3.2 ± 1.6
Protruding vulva (%)	0.0	5.6 ± 3.9
Adult death (%)	4.7 ± 2.3	12.9 ± 3.2
Eggs in uterus	12.6 ± 2.8	15.3 ± 6.8
Average brood size	312 ± 11.1	190 ± 79.5
Incidence of males (%)	0.0	4.9 ± 2.05

### Genetic interactions between the spindle checkpoint genes and *hcp-1*

To further analyze the *san-1 *function in *C. elegans *we used RNAi to identify additional genes that genetically interact with *san-1(ok1580)*. We were interested in determining if RNAi of specific gene products resulted in a more severe phenotype in the *san-1(ok1580) *background. This approach is similar in concept to a synthetic lethal screen, an established technique to identify genetic interactions in which the double mutant has a phenotype distinct or more severe than the phenotype(s) of the individual single mutants. The genes that we evaluated included those that are involved with kinetochore assembly or spindle checkpoint function and do not have severe embryonic lethality phenotypes. Thus, we analyzed *hcp-1*, *hcp-2*, the putative *bub-3 *homologue Y54G9A.6, the known spindle checkpoint genes, *mdf-1 *and *mdf-2*, and the predicted kinetochore protein *kbp-5 *[18]. The *hcp-1/2 *genes encode kinetochore-localized proteins required for kinetochore assembly, and animals only have a significant defect in viability when both HCP-1/2 are simultaneously depleted by RNAi [23,25]. The gene product of Y54G9A.6 has homology to the spindle checkpoint gene BUB-3 (see Additional file 1). BLASTp analysis of *C. elegans *Y54G9A.6 predicted gene product suggests there is homology with yeast BUB3 (E value = 4e-28), *Homo sapiens *BUB3 (E value = 3e-27), and *D. malanogaster *BUB3 (E value = 2e-74). The amino acid identity between the *C. elegans *Y54G9A.6 predicted gene product and BUB3 proteins is 44% for *H. sapiens*, 43% for *D. melanogastor *and 19% for *Saccharomyces cerevisiae*, as determined using the ClustalW software. Thus, the Y54G9A.6 gene will be referred to as *bub-3 *in this report. Other gene candidates likely to interact with *san-1*, such as other spindle checkpoint genes (*bub-1*), genes that encode products essential for kinetochore assembly and function (*hcp-3*, *knl-1*, *knl-3*, *cls-2*) and homologues to zw10 and rod (*czw-1*, *rod-1*), were not analyzed because RNAi of these gene products result in severe embryonic lethality, thus testing a synthetic lethal interaction with *san-1(ok1580) *is not possible. We chose to focus our analysis instead on genes *bub-3*, *mdf-1*, *mdf-2*, *hcp-1*, *hcp-2 *and *kbp-5*.

**Figure 2:**
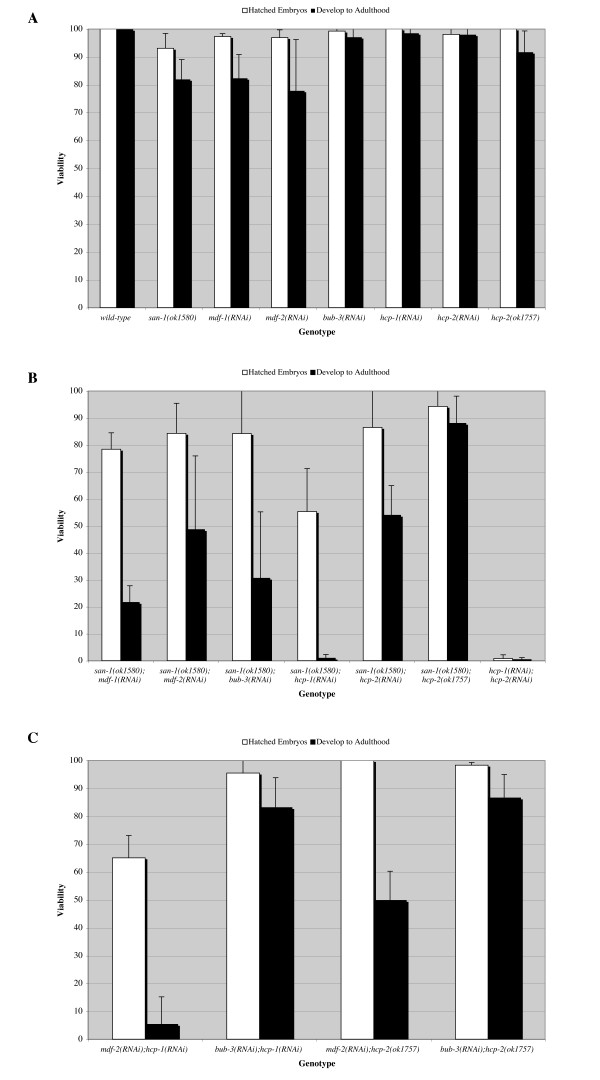
**Synthetic lethality of spindle checkpoint genes**. For graphs A, B, and C, the embryos, of the designated genotypes, were assayed for the ability to hatch (white filled bars) and the ability to develop to adulthood after three days on food (black filled bars). The *san-1(ok1580);hcp-2(ok1757) *animals were given four days to develop to adulthood. The data obtained is from four independent experiments with > 200 total animals assayed. Error bar is standard deviation.

A reduction in the spindle checkpoint genes (*san-1*, *mdf-1*, *mdf-2*, *bub-3*) or the kinetochore assembly genes (*hcp-1*, *hcp-2) *individually did not result in severe embryonic lethality and the majority of animals developed into adults (Figure 2A). The *mdf-1(RNAi) *and *mdf-2(RNAi) *animals had several phenotypes as adults including death due to vulva bursting or formation of a bag of worms (data not shown), which is consistent with what others have observed [18]. The *mdf-1(gk2) *deletion mutant has a significant embryo lethal phenotype [18], suggesting that the *mdf-1(RNAi) *acts as a null. In comparison to *san-1(ok1580) *animals, the *san-1(ok1580);bub-3(RNAi) *(P < .04), *san-1(ok1580);mdf-1(RNAi) *(P < .01) and *san-1(ok1580);hcp-1(RNAi) *(P < .0003) animals displayed a reduction in the ability for embryos to develop to adulthood (Figure 2B). Nearly all of the *san-1(ok1580);hcp-1(RNAi) *animals died as embryos or young larvae. The *san-1(ok1580);hcp-2(RNAi) *animals had a decrease in the ability to develop to adulthood, in comparison to *san-1(ok1580) *animals (P < .05), however the phenotype was not as a severe as the *san-1(ok1580);hcp-1(RNAi) *animals (Figure 2b). The *san-1(ok1580);mdf-2(RNAi) *had a reduced but not significant difference in viability (P = .073). We did not observe a viability defect in *san-1(ok1580);kbp-5(RNAi) *animals thus *kbp-5 *was not further analyzed (data not shown).

Because RNAi does not always completely remove all of a genes function, we used a genetic deletion of *hcp-2 *to gain a greater understanding of the genetic interaction between *hcp-2 *and *san-1*. We crossed the *hcp-2(ok1757) *and *san-1(ok1580) *animals to produce a *san-1(ok1580);hcp-2(ok1757) *animal. The *hcp-2(ok1757) *allele is a 1.281 kb deletion, which removes most of exon 5 resulting in a frame-shift; *hcp-2 *has 8 predicted exons (data not shown). We verified this deletion by DNA sequence analysis. We determined that 26.25% of the F2 offspring (n = 80) from a *san-1(ok1580) *and *hcp-2(ok1757) *cross had phenotypes including embryo lethality, larvae lethality, odd morphology and lethargy. The number of F2 progeny with abnormal phenotypes was higher than the expected 1/16 (6.25%) for a homozygous double mutant animal (*san-1(ok1580);hcp-2(ok1757))*, suggesting that some of the F2 progeny that are a combination of homozygous and heterozygous mutations for either *san-1(ok1580) *or *hcp-2(ok1757) *may have an abnormal phenotype. We genotyped 39 F2 animals, using single worm PCR to detect the *san-1 *and *hcp-2 *deletions, and determined that the homozygous double mutant (*san-1(ok1580);hcp-2(ok1757)*) and animals homozygous for the *san-1(ok1580) *deletion and heterozygous for the *hcp-2(ok1757) *deletion displayed embryonic and larval lethality phenotypes. The *hcp-2(ok1757) *animals appeared to be lethargic as adults, as noted by their reduction in motility, yet did not exhibit embryonic lethality and the majority reached adulthood three days after egg laying (Figure 2A). We isolated a *san-1(ok1580);hcp-2(ok1757) *double mutant and determined that the majority of the embryos hatched, yet only 7.3 % ± 2.4 of the larvae reached adulthood within three days at 20°C. However, if given four days to develop the majority of *san-1(ok1580);hcp-2(ok1757) *animals developed to adulthood (Figure 2b). Several of the *san-1(ok1580);hcp-2(ok1757) *animals had morphological defects suggesting that the double mutant had a more severe phenotype then the single mutants (data not shown). Our results indicate that the phenotype observed in *san-1(ok1580);hcp-2(ok1757) *is not identical to that observed in *san-1(ok1580);hcp-1(RNAi) *animals.

Nearly all of the *hcp-1(RNAi);hcp-2(RNAi) *embryos die during embryogenesis; however, over half of the *san-1(ok1580);hcp-1(RNAi) *or *mdf-2(RNAi);hcp-1(RNAi) *animals survive embryogenesis but die as larvae (Figure 2B, 2C). Thus, the embryo lethality of *san-1(ok1580);hcp-1(RNAi) *or *mdf-2(RNAi);hcp-1(RNAi) *animals is not as severe in comparison to the *hcp-1(RNAi);hcp-2(RNAi) *animals [23]. In comparison to *mdf-2(RNAi) *animals, the *mdf-2(RNAi);hcp-1(RNAi) *animals had a significant decrease in the ability to develop and survive to adulthood (P < .011). The *mdf-2(RNAi);hcp-2(ok1757) *animals also had a decrease in the ability to survive to adulthood (P < .025), however the viability defect was not as severe as that observed in *mdf-2(RNAi);hcp-1(RNAi) *animals (Figure 2C). Similar to *bub-3(RNAi) *or *hcp-1(RNAi) *animals, the *bub-3(RNAi);hcp-1(RNAi) *animals did not have a viability defect (Figure 2C). Furthermore, the *bub-3(RNAi);hcp-2(ok1757) *(Figure 2C) and *bub-3(RNAi);hcp-2(RNAi) *(data not shown) animals did not have viability defects. Using RT-PCR, in comparison to wild-type, a greater than 5 fold reduction in *bub-3 *transcript level (P < .029) was observed in *bub-3(RNAi) *animals, thus demonstrating RNAi efficiency. Together, these results suggest that *san-1 *genetically interacts with *bub-3*, *mdf-2, mdf-1, hcp-1 *and *hcp-2 *and that *C. elegans *requires both *hcp-1 *and either of the checkpoint genes, *san-1 *or *mdf-2 *but not *bub-3*. Furthermore, these results suggest that *hcp-1 *and *hcp-2 *have redundant but not identical functions.

### The *san-1(ok1580);hcp-1(RNAi) *animals develop abnormally

**Figure 3:**
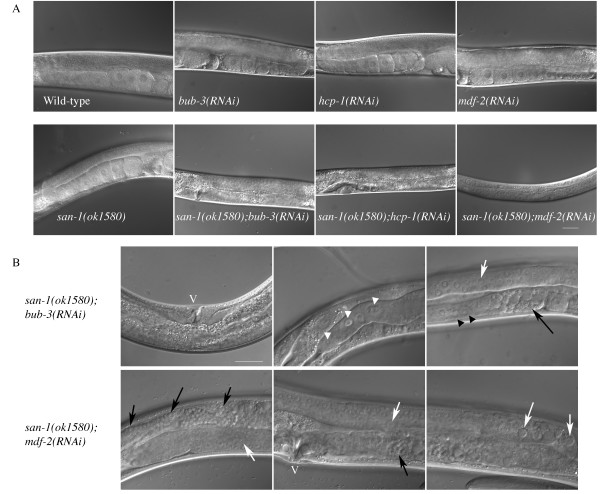
**Phenotype analysis and gonad morphology of *san-1(ok1580);bub-3(RNAi)*, *san-1(ok1580);hcp-1(RNAi)*, *san-1(ok1580);mdf-2(RNAi) *animals**. L1 larvae were grown at 20°C for three days before gonad morphology was assayed using DIC microscopy. Phenotypes observed include a larvae arrest or slow growth phenotypes (A). For *san-1(ok1580);bub-3(RNAi) *or *san-1(ok1580);mdf-2(RNAi) *animals phenotypes included abnormal cell morphology in the mitotic region of the gonad (white arrow head), abnormal cell morphology in the meiotic region of the gonad (white arrow), and abnormal oocyte morphology (B, black arrow head). Animals could produce sperm (B, black arrows), but the spermatheca often appeared abnormal as indicated by sperm localization. The position of the vulva is indicated by a V. Bar = 25 μm.

To further characterize the phenotype of the *san-1(ok1580);mdf-2(RNAi)*, *san-1(ok1580);bub-3(RNAi) *and *san-1(ok1580);hcp-1(RNAi) *animals, we analyzed the animals for developmental defects. The majority of animals, that did not die as embryos, arrested as larvae or had an abnormal gonad as adults (Figure 3, Table 2). First, the *san-1(ok1580);mdf-2(RNAi)*, *san-1(ok1580);bub-3(RNAi) *and *san-1(ok1580);hcp-1(RNAi) *animals had larvae arrest or slow growth phenotypes (Figure 3A). Additional defects observed in the *san-1(ok1580);mdf-2(RNAi) *or *san-1(ok1580);bub-3(RNAi) *animals included abnormal cell morphology in the mitotic region of the gonad, abnormal cell morphology in the meiotic region of the gonad, meiotic cells that had button like cell morphology reminiscent of apoptotic cells, and abnormal oocyte structure (Figure 3B). Some of the animals produced sperm but the spermatheca appeared to be abnormal in structure as determined by sperm localization (Figure 3B, black arrows). We also observed gonad migration defects (data not shown). The majority of *san-1(ok1580);hcp-1(RNAi) *animals died or arrested as larvae. Abnormal gonad morphology was not observed in the *bub-3(RNAi) *or *hcp-1(RNAi) *animals. Some of the *san-1(ok1580) *and *mdf-2(RNAi) *had abnormal gonad morphology (Table 2).

**Table 2:** Analysis of gonad morphology

Genotype	**Abnormal Gonad (%)**	**No.**
Wild-type	0.0	25
*bub-3(RNAi)*	0.0	25
*hcp-1(RNAi)*	0.0	33
*mdf-2(RNAi)*	37.8	37
*san-1(ok1580)*	10.3	29
*san-1(ok1580);bub-3(RNAi)*	84.8	33
*san-1(ok1580);hcp-1(RNAi)*	100.0	9
*san-1(ok1580);mdf-2(RNAi)*	94.4	36

Animals were assayed using DIC microscopy and scored for abnormal gonad development, which included the absence of oocytes, abnormal gonad morphology, and abnormal cell structure in the mitotic or meiotic region of the gonad. The total number of animals analyzed (No.).

**Figure 4:**
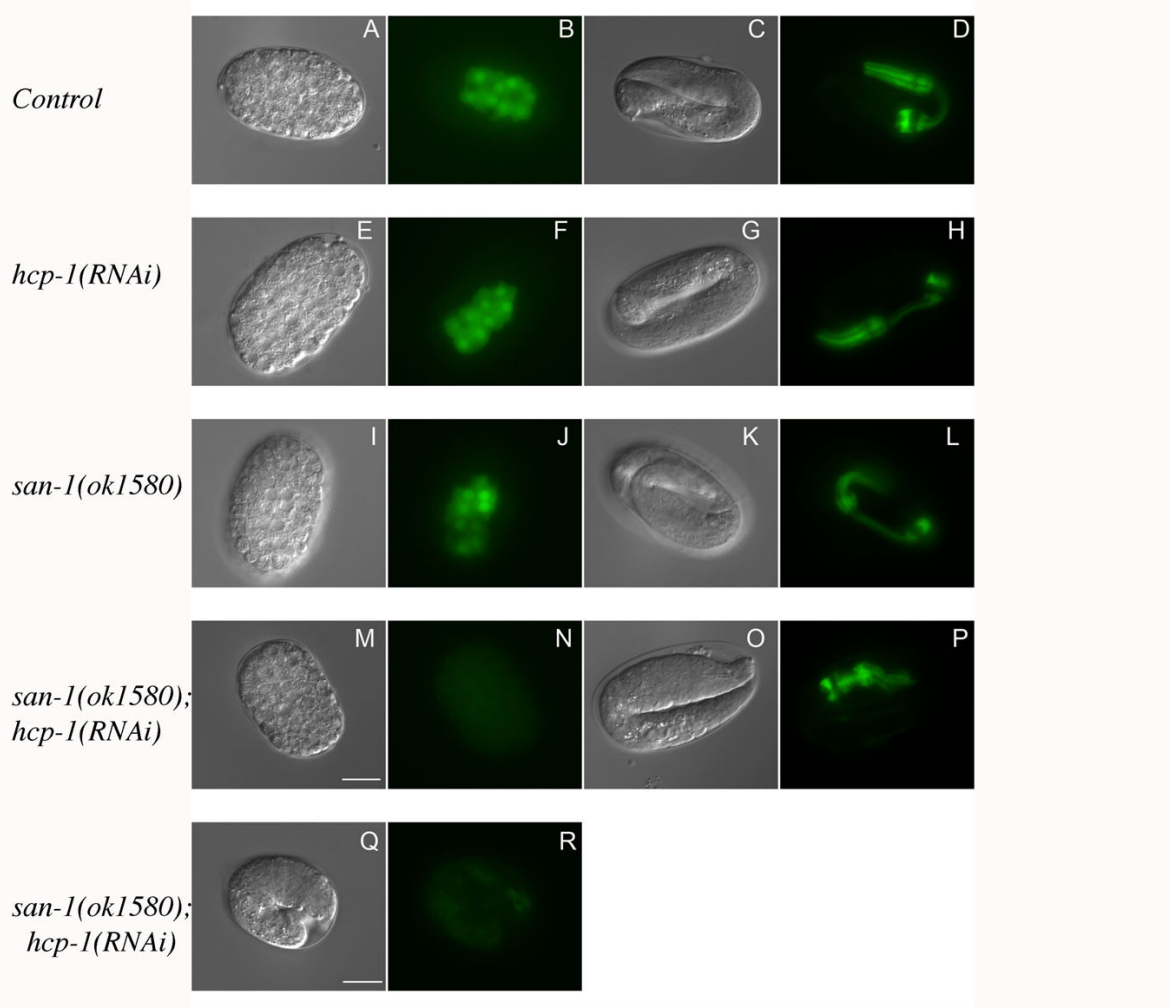
**The *san-1(ok1580);hcp-1(RNAi) *animals have abnormal development**. DIC microscopy and the MYO-2::GFP fusion protein, which is expressed in cells differentiating to the pharynx, was used to assay pharynx development. Pharynx morphology, in control, *hcp-1(RNAi)*, *san-1(ok1580)*, and *san-1(ok1580);hcp-1(RNAi) *embryos was assayed at early (A, B, E, F, I, J, M, N) and late embryogenesis (C, D, G, H, K, L, O, P, Q, R). Bar = 15 μm.

The *hcp-1(RNAi);hcp-2(RNAi) *animals die as embryos whereas many of the *san-1(ok1580);hcp-1(RNAi) *embryos hatch and later die as young larvae (Figure 2). To determine if the *san-1(ok1580);hcp-1(RNAi) *embryos have developmental defects we used the pharynx as a marker for developmental progression, by analyzing the expression of *myo-2::GFP*. Briefly, *myo-2 *encodes a muscle-type specific myosin heavy chain isoform and is expressed during embryogenesis in the primordial pharyngeal muscle cells. Similar to the control embryos (Figure 4A–D), the *hcp-1(RNAi) *(Figure 4E–H) and *san-1(ok1580) *(Figure 4I–L) animals had normal pharynx development. However, the *san-1(ok1580);hcp-1(RNAi) *embryos had either a reduction in *myo-2::GFP *(Figure 4M–N, Q–R) or abnormal pharynx structure (Figure 4O–P). Together, these results indicate that the *san-1(ok1580);hcp-1(RNAi) *embryos have developmental defects.

### Spindle checkpoint activity

Our data suggest that the development of *san-1(ok1580) *animals depends upon *bub-3 *and *hcp-1*, however it is not clear if, like the *san-1*,*mdf-1 *and *mdf-2 *genes, the *bub-3 *and *hcp-1 *gene products function in the spindle checkpoint pathway. It is known that blastomeres of wild-type embryos exposed to oxygen deprivation (anoxia) reversibly arrest cell cycle progression [21,29]. Unlike wild-type embryos, *san-1(RNAi) *and *mdf-2(RNAi) *embryos are sensitive to the microtubule disrupting environments of anoxia or nocodazole treatment. Specifically, *san-1(RNAi) *and *mdf-2(RNAi) *embryos exposed to anoxia have a significantly reduced number of metaphase blastomeres and an increase in anaphase bridging and chromosomal abnormalities; supporting the idea that *san-1 *and *mdf-2 *function as spindle checkpoint genes [21,22]. Similar to what is known regarding the *san-1(RNAi) *embryos, the *san-1(ok1580) *embryos exposed to anoxia had a reduced survival rate (Table 3). The *bub-3(RNAi) *embryos exposed to anoxia also had a reduced survival rate in comparison to wild-type animals suggesting that *bub-3 *functions as a spindle checkpoint protein in developing embryos. The *hcp-1(RNAi) *embryos were not sensitive to anoxia (Table 3). Additionally, we did not observe a sensitivity to anoxia in *hcp-2(RNAi) *or *hcp-2(ok1757) *embryos (data not shown). These results suggest that *hcp-1 *and *hcp-2 *are not required for anoxia induced metaphase arrest and do not function as a spindle checkpoint protein.

**Table 3:** Anoxia sensitivity to assay spindle checkpoint activity

**Genotype**	**Anoxia viability **^a^		
		
	**Hatch (%)**	**Adulthood (%)**	**No.**
Wild-type	99.2 ± 1.1	99.0 ± 1.1	511
*san-1(ok1580)*	77.3 ± 8.4	42.7 ± 21.4 (P < .014)	482
*bub-3(RNAi)*	90.2 ± 3.1	81.0 ± 12.0 (P < .053)	481
*hcp-1(RNAi)*	92.6 ± 8.9	92.3 ± 8.5	230

^a^Animals were assayed for sensitivity to 1 day of anoxia by determining the percentage of anoxia exposed embryos that are able to hatch and develop to adulthood. Four independent experiments with a total number of embryos assayed (No.).

### Analysis of *san-1(ok1580);hcp-1(RNAi) *mitotic blastomeres

**Figure 5:**
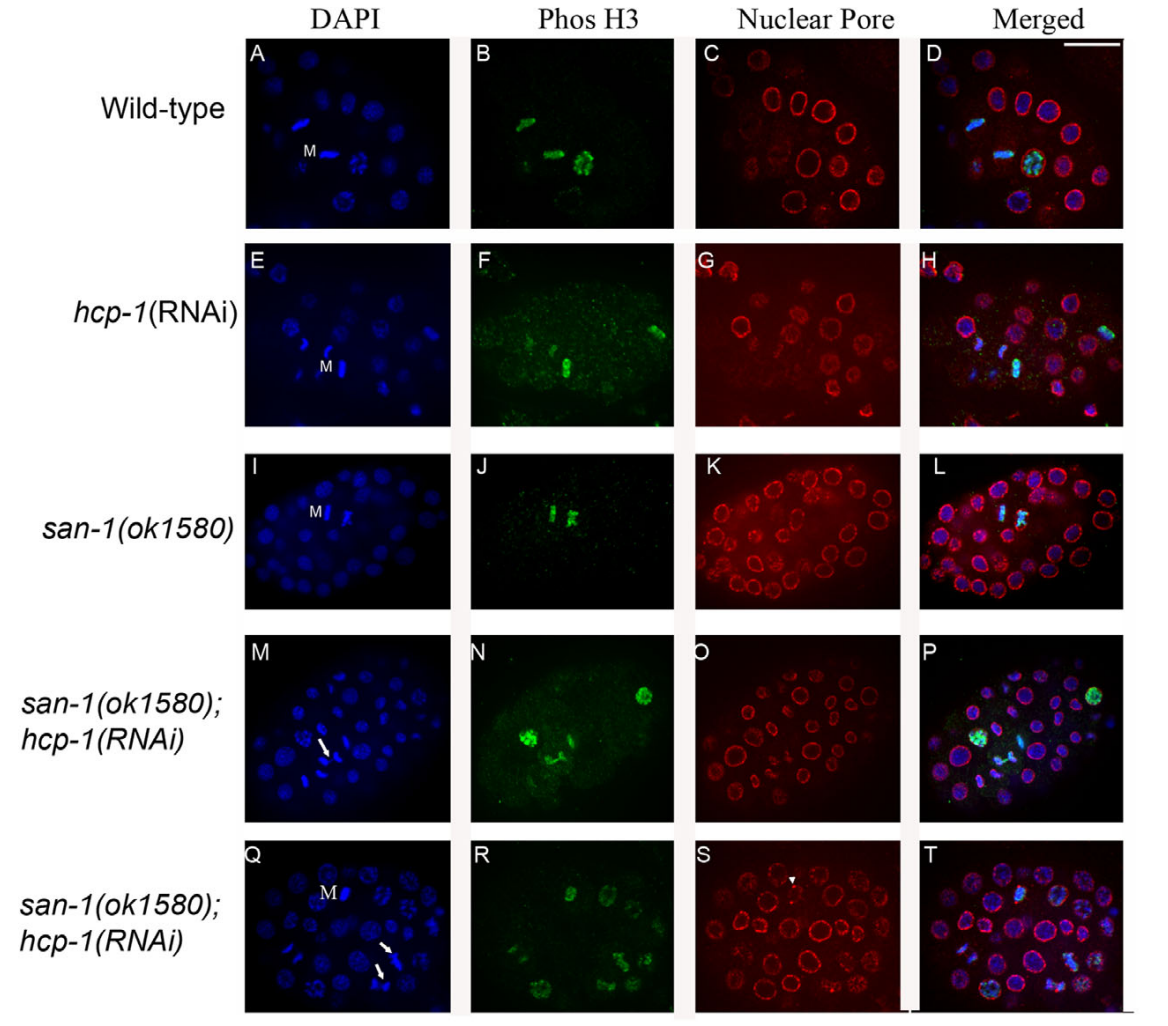
**The *san-1(ok1580);hcp-1(RNAi) *embryos contain blastomeres with abnormal chromosomes**. The Phos H3 antibody recognizes the phosphorylated form of Histone H3 specific for mitotic cells (prophase to anaphase) and the mAb414 recognizes the nuclear pore complex. Images were collected using a spinning disc confocal microscope. The arrows point to abnormal mitotic nuclei. Arrowhead points to a metaphase blastomere that display aggregates recognized by mAb414 in a metaphase blastomere. M denotes metaphase blastomeres. Two typical *san-1(ok1580);hcp-1(RNAi) *embryos are shown. Images depict a single focal plane. Bar = 10 μm.

To gain a greater understanding of the functional relationship between the *hcp-1 *and *san-1 *gene products in developing embryos we determined if chromosome loss was occurring in the *san-1(ok1580);hcp-1(RNAi) *embryos. We analyzed chromosome structure in mitotic blastomeres from wild-type, *hcp-1(RNAi)*, *san-1(ok1580)*, and *san-1(ok1580);hcp-1(RNAi) *embryos collected, fixed and stained as previously described [22]. We used mAb414, which recognizes the nuclear pore complexes and anti-PhosH3, which recognizes the phosphorlyated form of histone H3 to distinguish mitotic blastomeres [22,29-31]. We found that unlike wild-type, *san-1(ok1580)*, and *hcp-1(RNAi) *embryos (Figure 5A–L), the *san-1(ok1580);hcp-1(RNAi) *embryos had a significant increase in abnormal nuclei (44%, n = 50) (Figure 5M–T). These abnormalities included abnormal mitotic structure, lagging chromosomes, and anaphase bridging (Figure 5M, 5Q, arrows). These defects were also observed in young embryos (2–4 cell embryos; 15.4% (n = 13)), indicating that the chromosome segregation defects occur early in embryo development.

In young wild-type embryos (less than 30 blastomeres) the nuclear pore complexes detected by mAb 414 do not diminish during mitosis [31]. In contrast, embryos with greater than 30 blastomeres, the nuclear pore complexes detected by mAb 414 diminish from prometaphase to anaphase. Similar to wild-type embryos (Figure 5A–D), with greater than 30 blastomeres, the nuclear pore complexes, detected by mAb414, were diminished in metaphase blastomeres of *hcp-1(RNAi) *(Figure 5E–H), and *san-1(ok1580) *embryos (Figure 5I–L). However, in *san-1(ok1580);hcp-1(RNAi) *embryos with greater than 30 blastomeres, the nuclear pore complex, detected by mAb414, were in small aggregates surrounding the metaphase plates (72%, n = 11) (Figure 5Q–T, 5S arrow head). This phenotype can only be observed in the older embryos, since the younger embryos do not have diminished nuclear pore complexes. The accumulation of abnormal nuclei and aggregates of the nuclear pore complex suggests that progression through mitosis was aberrant in the *san-1(ok1580);hcp-1(RNAi) *embryos.

**Figure 6:**
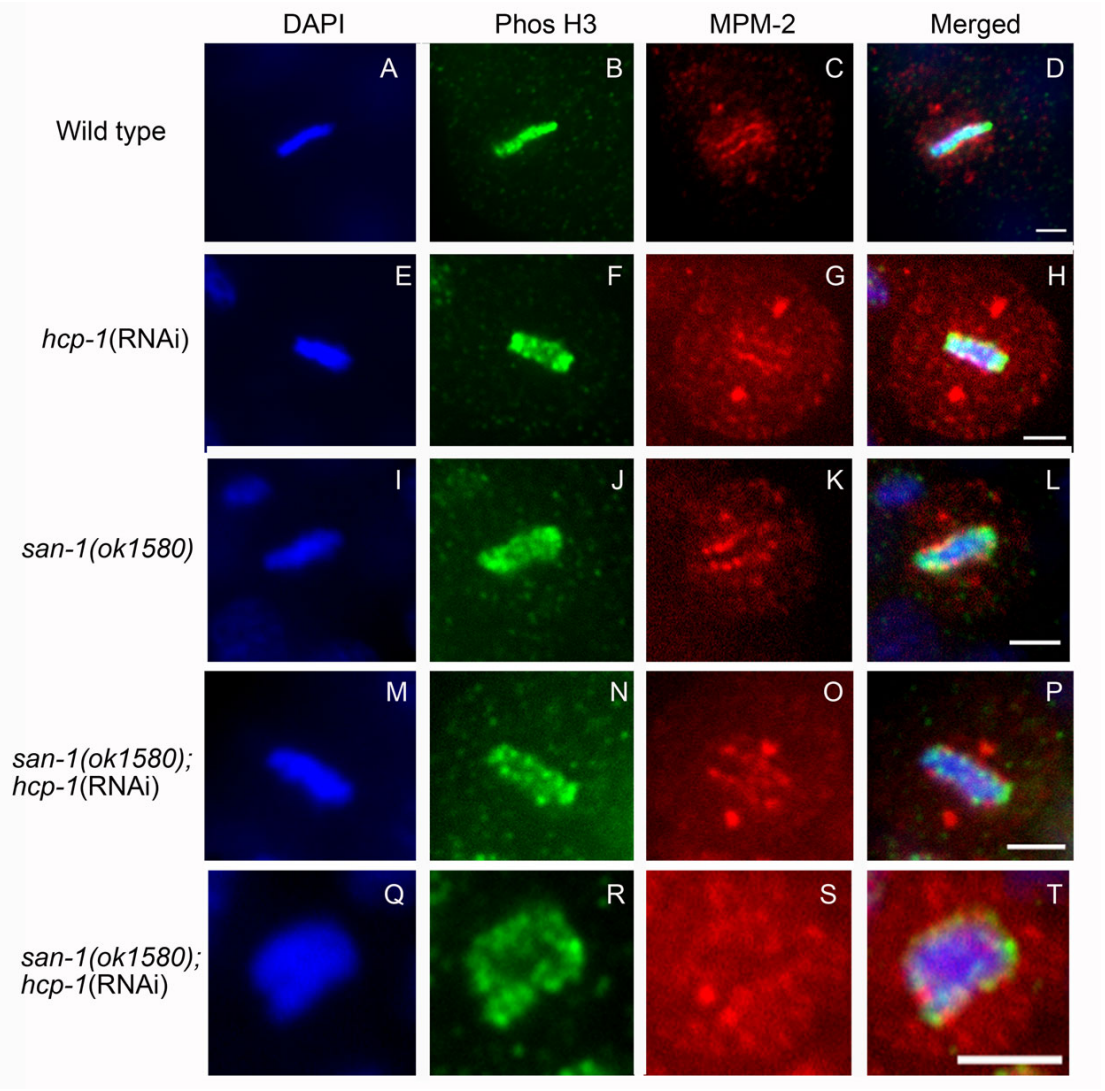
**The *san-1(ok1580);hcp-1(RNAi) *embryos have normal and abnormal kinetochore and centriole structure**. Analysis of metaphase blastomeres of wild-type (A-D), *hcp-1(RNAi) *(E-H), *san-1(ok1580) *(I-L) and *san-1(ok1580);hcp-1(RNAi) *embryos (M-T) was assayed. The MPM-2 antibody recognizes mitotic proteins located at the kinetochore and centrioles. Two typical *san-1(ok1580);hcp-1(RNAi) *metaphase blastomeres are shown. The metaphase blastomere shown in Q-T is of abnormal structure. Bar = 10 μm.

To gain a greater understanding of mitotic progression in the *san-1(ok1580);hcp-1(RNAi) *embryos we analyzed chromosome, kinetochore and spindle microtubule structure using indirect immunofluorescence. We first stained embryos with MPM-2, an antibody that recognizes mitotic proteins localized to the kinetochore and centrosomes [32]. The kinetochore structure in *san-1(ok1580) *and *hcp-1(RNAi) *embryos was similar to that of wild-type embryos (Figure 6C,G,K). However, the *san-1(ok1580);hcp-1(RNAi) *embryos contained both normal and abnormal kinetochore structure, as determined by the presence or absence of the two-line formation along the sister chromatids (Figure 6O, 6S). The abnormal kinetochore structure correlated with abnormal mitotic structure.

**Figure 7:**
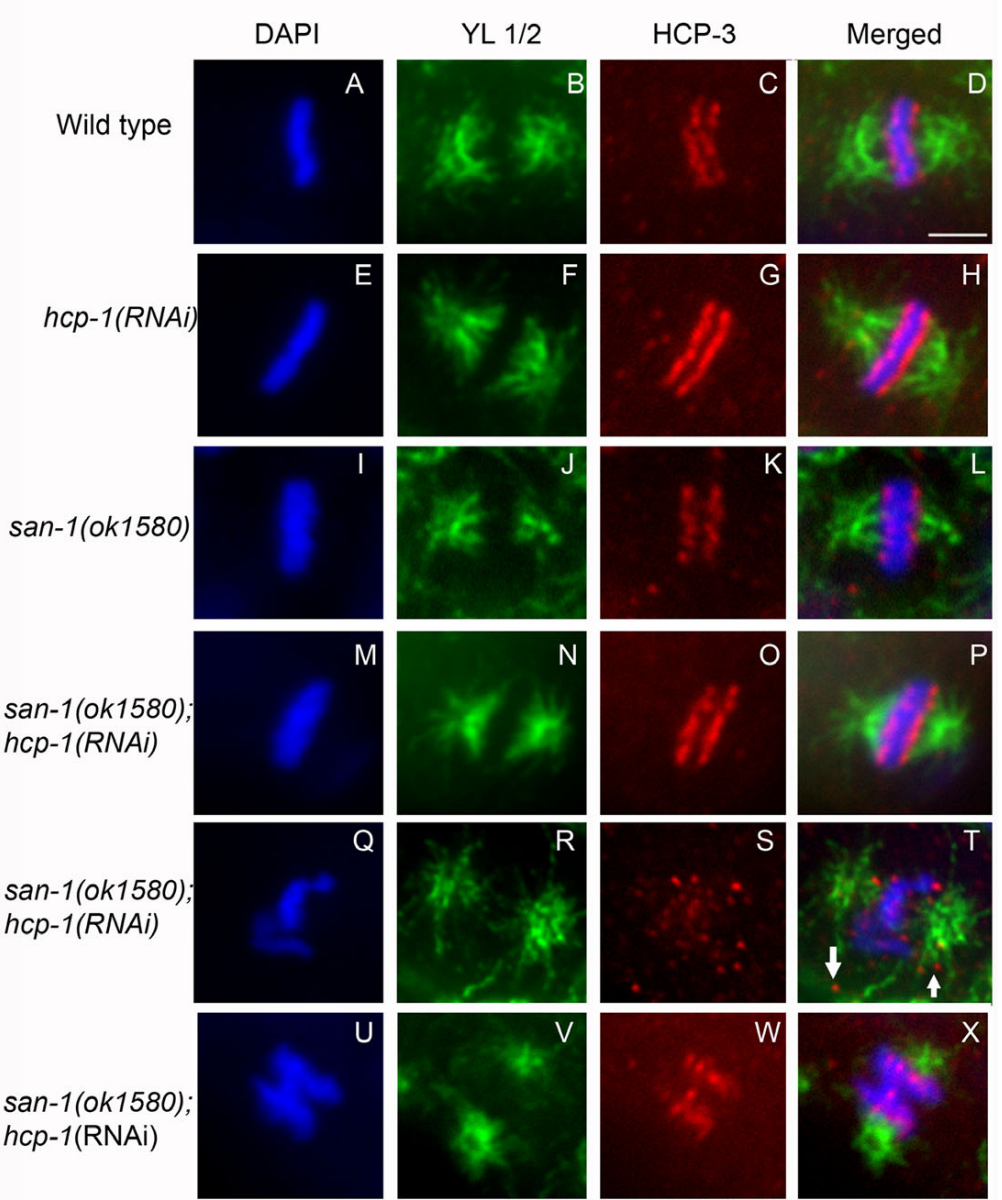
**The *san-1(ok1580);hcp-1(RNAi) *mitotic blastomeres have normal and abnormal centromere and spindle structure**. Analysis of metaphase blastomeres of wild-type (A-D), *hcp-1(RNAi) *(E-H), *san-1(ok1580) *(I-L) and *san-1(ok1580);hcp-1(RNAi) *embryos (M-X) was assayed. Anti-HCP-3 recognizes the centromeric histone HCP-3 and YL1/2 antibody recognizes microtubules. Three typical *san-1(ok1580);hcp-1(RNAi) *metaphase blastomeres are shown. The metaphase blastomeres shown in Q-X are of abnormal structure. Images were collected using a spinning disc confocal microscope. Arrow points to HCP-3 not associated with the chromosomes. Bar = 2 μm.

We used anti-HCP-3 and YL1/2 to analyze the localization of the centromeric histone HCP-3 (CENP-A) and spindle microtubules, respectively [23,26]. HCP-3 is at the top of the kinetochore assembly pathway [23,25]. The centromere structure and spindle microtubules in *san-1(ok1580) *and *hcp-1(RNAi) *embryos were similar to that of wild-type embryos (Figure 7A–L). The *san-1(ok1580);hcp-1(RNAi) *blastomeres with normally formed metaphase plates had normal centromere and microtubule structure (Figure 7M–P). The *san-1(ok1580);hcp-1(RNAi) *blastomeres with abnormal chromosome structure contained HCP-3 localized with chromosomes (Figure 7U–X); yet in some blastomeres HCP-3 was not always associated with the chromosomes (Figure 7Q–T). In these abnormal blastomeres the microtubule structure was abnormal (Figure 7Q–X). Embryos analyzed for these experiments contained ~20–30 blastomeres so it is possible that these defects are due to altered gene expression resulting from chromosome segregation defects.

Given the presence of anaphase bridging, lagging chromosomes, and abnormal chromosome structure observed in the *san-1(ok1580);hcp-1(RNAi) *animals, it is likely that the blastomeres were transitioning through mitosis abnormally. To determine if this occurred *in vivo *we produced a *san-1(ok1580)*;*tbg-1::GFP;pie-1::GFP::H2B *strain (PM115 strain, histone and gamma-tubulin GFP-tagged proteins) for live cell imaging using a spinning disc confocal microscope. The *san-1(ok1580);hcp-1(RNAi);tbg-1::GFP;pie-1::GFP::H2B *embryos contained blastomeres with abnormal mitotic progression. Analysis of a typical *san-1(ok1580);hcp-1(RNAi) *embryo showed one blastomere which progressed to anaphase with normal chromosome segregation, and another blastomere with abnormal chromosome segregation leading to anaphase bridging (see Additional file 2). These results further demonstrated that abnormal chromosome segregation occurred in the *san-1(ok1580);hcp-1(RNAi) *embryos.

**Figure 8:**
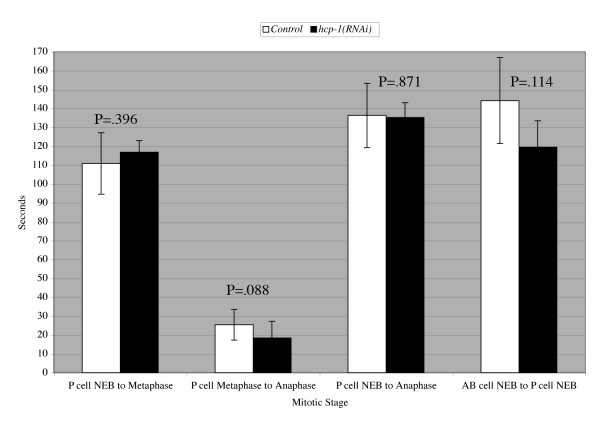
**The *hcp-1(RNAi) *2-cell embryo does not have a significant difference in mitotic timing in comparison to wild-type 2-cell embryos**. Live cell imaging with a spinning disc confocal microscope was used to analyze mitotic progression in *hcp-1(RNAi)*;*tbg-1::GFP;pie-1::GFP::H2B *and control animals. The AB and P cells of a 2-cell embryo were analyzed. In the P cell of a wild-type and *hcp-1(RNAi) *2-cell embryo the time it took (seconds) to transition from nuclear envelope break down (NEB) to formation of the metaphase plate, formation of the metaphase plate to anaphase onset and NEB to anaphase onset was determined. The time between NEB of the AB cell and NEB of the P cell was also determined. Each experiment was conducted at 21°C (n = 7 embryos). The P value was determined using a standard student t test.

Thus far, we have shown that the *san-1(ok1580);hcp-1(RNAi) *embryos abnormally segregate chromosomes which likely leads to developmental defects and lethality. The *hcp-1(RNAi) *embryos have not been shown to have chromosome segregation issues that lead to increased lethality. One possible reason for the synthetic lethal interaction between *san-1 *and *hcp-1 *is that loss of *san-1 *amplifies a subtle *hcp-1 *defect. Given the defect in progression through mitosis observed with mAb 414, timing of mitotic progression is possibly perturbed when *hcp-1 *is absent and loss of *san-1 *increases this defect. Therefore, we conducted live cell imaging to compare mitotic progression in *hcp-1(RNAi)*;*tbg-1::GFP;pie-1::GFP::H2B *and control animals. We analyzed mitosis in the AB and P cell blastomeres of a 2-cell embryo. We determined the time it took to transition from nuclear envelope break down (NEB) to formation of the metaphase plate, metaphase to anaphase onset and NEB to anaphase onset in the P cell of a wild-type and *hcp-1(RNAi) *2-cell embryo. We also determined the time between NEB of the AB cell and NEB of the P cell. We found that the mitotic progression of *hcp-1(RNAi) *embryos analyzed at 21°C did not have any chromosomal defects and the time it took to progress through mitosis was not significantly different in comparison to control embryos (Figure 8). Together, our data indicates that *hcp-1(RNAi) *animals require a functional spindle checkpoint for normal development.

## Discussion

### Genetic interactions with *san-1*

The use of synthetic lethal screens is a genetic tool to elucidate genes that interact in similar cellular functions or pathways. Thus, when a genetic mutant does not have a severe phenotype the use of a synthetic lethal screen to identify double mutants that have a phenotype distinct or more severe then the phenotype(s) observed for the individual single mutants often will aid in further understanding the function of the gene products. To analyze *san-1 *function in *C. elegans *embryos we used RNAi to identify genes that could genetically interact with *san-1(ok1580)*. We determined that *san-1(ok1580);hcp-1(RNAi)*, *san-1(ok1580);bub-3(RNAi), san-1(ok1580);mdf-1(RNAi)*, and *san-1(ok1580);mdf-2(RNAi) *had a reduced ability to survive. The genetic interaction between *san-1*, *mdf-1*, and *mdf-2 *is expected, since these genes are thought to function in the spindle checkpoint pathway in mitotic and meiotic cells [18-21]. The predicted *C. elegans bub-3 *gene product has high homology to BUB-3 from other systems and *bub-3(RNAi) *embryos are more sensitive to the microtubule disrupting process of anoxia exposure, supporting the idea that the Y54G9A.6 gene encodes the *bub-3 *spindle checkpoint gene.

The genetic interaction between *hcp-1/2 *and the spindle checkpoint genes is likely to be more complex. The viability of *san-1(ok1580);hcp-1(RNAi) *and *mdf-2(RNAi);hcp-1(RNAi) *animals was severely compromised, suggesting that HCP-1 interacts with MDF-2 and SAN-1. However, the *bub-3(RNAi);hcp-1(RNAi) *animals did not have a significant decrease in survival rate. This could be due to BUB-3 and HCP-1 not involved together in a specific function. The *san-1(ok1580);hcp-2(ok1757) *animals have embryonic and larvae lethal phenotypes. We isolated a *san-1(ok1580);hcp-2(ok1757) *double mutant and determined that it had more severe phenotypes than that observed in the single mutants; this suggests that *hcp-2 *and *san-1 *genetically interact. However, the phenotype of the *san-1(ok1580);hcp-2(ok1757) *double mutants was not as severe as that observed *san-1(ok1580);hcp-1(RNAi) *animals, suggesting that HCP-1 and HCP-2 are not be completely redundant. HCP-1 and HCP-2 are 54% similar mostly at their N- and C-termini [23]. Although HCP-1 shares some similarity to human CENP-F, primarily in a tandem repeat present in both HCP-1 and CENP-F, HCP-2 lacks significant similarity to CENP-F [23]. Thus, it is possible that HCP-1 and HCP-2 have overlapping and distinct functions. It will be of interest to determine the specific molecular interactions between HCP-1, HCP-2, SAN-1 and MDF-2.

### Functional analysis of spindle checkpoint genes and *hcp-1 *in gonad development and mitosis

The majority of *san-1(ok1580);hcp-1(RNAi) *animals had severe developmental defects including embryo and larvae lethality. The viability defects observed are likely due to abnormal chromosome segregation, which will in turn compromise cellular structure and function. The *san-1(ok1580);bub-3(RNAi) *and *san-1(ok1580);mdf-2(RNAi) *animals had gonad defects. We and others have shown that *san-1(ok1580) *and *mdf-2(RNAi) *animals have low level gonad defects [18,19]; yet these defects are much more severe if two spindle checkpoint genes are reduced. We observed a reduction in brood size for *san-1(ok1580) *hermaphrodites and others have shown that *mdf-2*(av14) mutants have a reduced brood size, further supporting the role the checkpoint genes have in germline function [19]. Others have shown that *hcp-1(RNAi);hcp-2(RNAi) *animals also have meiotic defects, supporting the idea that *hcp-1 *has a role in meiosis [27]. The exact molecular function the spindle checkpoint proteins and HCP-1/2 have in meiosis needs to be further investigated.

The *san-1(ok1580);hcp-1(RNAi) *embryos have severe chromosome segregation defects, and on average 55.3% of the embryos die and approximately 0.9% of the animals reach adulthood. Thus, in *san-1(ok1580);hcp-1(RNAi) *embryos, embryogenesis can progress, but the ability to produce viable adults is severely compromised. We used *myo-2::GFP *to analyze the structure of the developing pharynx in the *san-1(ok1580);hcp-1(RNAi) *embryos and found that there was not only abnormal pharynx morphology but we often observed a reduction in the *myo-2::GFP *in the embryos. It is not known if the reduction in *myo-2::GFP *in the *san-1(ok1580);hcp-1(RNAi);myo-2::GFP *animals is due to abnormal differentiation, the loss of *myo-2::GFP *DNA or abnormal regulation of the *myo-2::GFP*. An increase in genome loss due to chromosome segregation issues could result in either of these possibilities.

Closer examination of the chromosomes, nuclear pore proteins, centromere, kinetochore, and microtubules indicates that the *san-1(ok1580);hcp-1(RNAi) *embryos have chromosome segregation defects leading to abnormal nuclei, anaphase bridging and lagging chromosomes. We determined that the nuclear pore complexes, detected by mAb414, formed small aggregates surrounding the metaphase plates in *san-1(ok1580);hcp-1(RNAi) *embryos, indicating that the nuclear membrane pore complexes are not completely breaking down. This may indicate that SAN-1 and HCP-1 influences additional mitotic events in addition to chromosome segregation. The centromeric protein HCP-3 was observed to have a normal or an abnormal localization pattern in the *san-1(ok1580);hcp-1(RNAi) *blastomeres. The abnormal localization of HCP-3 can be interpreted in several ways. First, it could be due to SAN-1 and HCP-1 directly regulating the localization of HCP-3 to the region that marks the centromere, however this seems unlikely since the kinetochore assembly pathway clearly places HCP-3 upstream of HCP-1 and SAN-1. Alternatively, the abnormal HCP-3 localization could be due to chromosome segregation defects causing loss of chromatin that marks the centromere. Finally, it is possible that the segregation defects result in chromatin fragments that HCP-3 associates with. Further studies would be required to determine the mechanism leading to abnormal HCP-3 localization.

In *san-1(ok1580);hcp-1(RNAi) *embryos, the kinetochore proteins recognized by the MPM-2 antibody were not associated with the chromosomes when the chromosome structure was aberrant. Furthermore, the microtubule structure was altered in blastomeres with abnormal chromosome structure. Together, these data suggests that in the *san-1(ok1580);hcp-1(RNAi) *blastomeres the kinetochore and microtubule interaction is sometimes, but not always, compromised. Given the increase in anaphase bridges, lagging chromosomes and abnormal mitotic nuclei, the microtubule and kinetochore interaction in *san-1(ok1580);hcp-1(RNAi) *animals is likely not sufficient for proper chromosome segregation.

Others have shown using mammalian cell culture (HeLa cells) that there is a prolonged mitosis in CENP-F (RNAi) cells and this prolonged mitosis is dependent upon the spindle checkpoint gene BUBR1 [33]. In *C. elegans hcp-1(RNAi);hcp-2(RNAi) *blastomeres take longer to progress through mitosis [20]. Our data suggest that *hcp-1(RNAi) *blastomeres of embryos analyzed at 21°C did not take longer to progress from prometaphase to metaphase, in comparison to control blastomeres, suggesting that mitotic timing of *hcp-1(RNAi) *blastomeres is normal as long as *hcp-2 *gene product is present.

CENP-F is required for chromosome alignment [34] and silencing of CENP-F in HeLa cells activates the spindle checkpoint [33]. The misalignment defect is thought to be due to kinetochores that do not correctly biorient rather than a defect in microtubule and kinetochore attachment [33]. Our results support the idea that in *hcp-1(RNAi) *animals the spindle checkpoint is being activated to ensure proper chromosome segregation and genome defects will occur without normal spindle checkpoint function; specifically SAN-1 and MDF-2 function are required. Given that the involvement of the spindle checkpoint in tumor progression, it is of interest to fully understand the genes products that interact with the spindle checkpoint pathway to ensure genome fidelity and maintenance.

## Conclusion

In this report we identified genes that genetically interact with the spindle checkpoint gene *san-1 *(mad-3 homologue). We show that the spindle checkpoint genes, *san-1 *and *mdf-2*, not only interact with one another but also interact with the CENP-F homologue *hcp-1*. The *bub-3 *spindle checkpoint gene does not genetically interact with *hcp-1*; suggesting that *hcp-1 *only interacts with a subset of the spindle checkpoint.

Detailed phenotype analysis of the *san-1(ok1580);hcp-1(RNAi) *embryos demonstrates that *C. elegans *embryos require *san-1 *and *hcp-1 *for proper chromosome segregation. Furthermore, gonad morphological defects were observed in *san-1(ok1580);mdf-2(RNAi) *and *san-1(ok1580);bub-3(RNAi) *animals further supporting a functional role of the spindle checkpoints in meiosis. We provide evidence to suggest that the *hcp-1 *and *hcp-2 *gene products may have overlapping but also distinct functions in *C. elegans*. Together, our data suggest that in the *C. elegans *embryo, HCP-1 functions in proper alignment and segregation of chromosomes and these functions are dependent upon elements of the spindle checkpoint pathway but not all of the spindle checkpoint proteins. Given the important role the spindle checkpoint and kinetochore proteins have in chromosome segregation and cell cycle progression, it is of great interest to characterize these gene products in developing organisms such as *C. elegans*.

## Materials and methods

### Strains and growth conditions

The wild-type Bristol strain (N2) and mutant strains were cultured on NGM plates seeded with *E. coli *(OP50) and raised at 20°C as described (Sulston and Hodgkin, 1988). The following strains were obtained from the *Caenorhabditis elegans *Genetics Center: RB1391(*san-1(ok1580)*), PD4790 (*myo-2::GFP*), TH32 (*tbg-1::GFP, pie-1::GFP::H2B*), and RB1492 (*hcp-2(ok1757)*). The *Caenorhabditis elegans *Gene Knockout Consortium (Oklahoma Medical Research Foundation) produced the *san-1(ok1580) *and *hcp-2(ok1757) *deletion alleles. The *hcp-2(ok1757) *mutant (strain RB1492) was back-crossed to wild-type animals three times (strain PM117). The *san-1(ok1580) *allele was crossed, using standard genetic techniques, to produce the following strains: PM107 (*san-1(ok1580)*;*myo-2::GFP)*, PM115 (*san-1(ok1580)*;*tbg-1::GFP;pie-1::GFP::H2B)*, and PM116 (*san-1(ok1580);hcp-2(ok1757)*). The *san-1(ok1580) *allele was backcrossed into wild-type background once. The *san-1(ok1580) *and *hcp-2(ok1757) *alleles were genotyped by single worm PCR with the following primers: *san-1 *forward primer (CGC TTA AAG CTT GAT CAA CTT CTCG), *san-1 *reverse primer (GCT AGT GAT TTC TCC TCC GTT TTC TCA), *hcp-2 *forward primer (ACT CTG AAG TCG GAA CAT GAA ATT), and *hcp-2 *reverse primer (TGA AGA GCC TTC TGT GCA AA). DNA sequence analysis to verify the deleted region of the *san-1(ok1580) *and *hcp-2(ok1757) *strains was conducted using standard molecular techniques.

### Analysis of BUB-3 protein sequence

The BUB-3 protein from the yeast *Saccharomyces cerevisiae *was used to conduct BLASTp against the *C. elegans*, *Homo sapiens *and *D. melanogastor *genome to identify the most identical protein for each species. The primary amino acid sequences were aligned using ClustalW and processed using the LaserGene sequence analysis software package (DNASTAR). Clustal analysis between the *C. elegans *Y54G9A.6 gene product (referred to as BUB-3) and the *S. cerevisiae, H. sapiens *and *D. melanogastor *BUB-3 proteins was conducted to determine percent identity between the proteins.

### Phenotype analysis of *san-1(ok1580)*

For all experiments animals were kept at 20°C and grown on *E. coli *(OP50). To assay for the high incidence of males (*him*) phenotype several hermaphrodites, grown in the absence of males, were allowed to lay eggs for several hours and the percent of male progeny was determined (for wild-type and *san-1(ok1580)*, n = 244, n = 325, respectively). The *san-1(ok1580) *animals were assayed for larval arrest and slow growth phenotypes (for wild-type and *san-1(ok1580)*, n = 210, n = 218, respectively). To determine if larval arrest or slow growth was observed we collected a synchronized population of L1 larvae and placed on OP50 NGM plates at 20°C for three to five days. We quantified the number of animals that developed to adulthood within three days, animals that developed to adulthood within three to five days (slow growth) and those that remained arrested as larvae (larval arrest). To assay for adult death phenotype, animals were grown for the first 5 days of adulthood and analyzed every day for death or survivorship (for wild-type and *san-1(ok1580)*, n = 210, n = 207, respectively). Hermaphrodites were moved daily to fresh plates and lost worms were eliminated from analysis. The average brood size was determined by quantifying the number of offspring individual hermaphrodites produced (for wild-type and *san-1(ok1580)*, n = 5, n = 10, respectively). To quantify the number of eggs within the uterus we collected 1-day old adults, placed on an agar pad and visualized using a Zeiss compound microscope; the number of eggs within the uterus was determined (for wild-type and *san-1(ok1580)*, n = 16). To assay for protruding vulva phenotype we collected 1-day old adults and counted the number of adult animals that had a protruding vulva (for wild-type and *san-1(ok1580)*, n = 210, n = 207, respectively). The p values (P) were determined using standard student t test.

### Synthetic lethal assays

RNA interference (RNAi) was used to decrease the expression of specific genes. For all experiments, the methodology was similar to that previously described (Hajeri et al). Briefly, a synchronous population of L1 larvae (N2, *san-1(ok1580)*) were grown to adulthood on NGM plates supplemented with 200 μg/ml ampicillin, 12.5 μg/ml tetracycline and 1 mM IPTG, seeded with a bacterial strain expressing dsRNA specific for *mdf-2*, *mdf-1*, *bub-3*, *hcp-1*, *hcp-2 *or control food [35]. Several adults were then placed on a secondary RNAi plate and allowed to lay eggs for 3–4 hours. The adults were removed and the embryos were grown at 20°C and assayed for embryonic lethality, larvae lethality, and morphological defects as seen by Nomarski microscopy using a motorized Zeiss Axioskop and imaged using Openlab 3.17. For each viability experiment at least three independent assays were conducted. The P-value (P) was determined using a standard student t-test. In addition to using the RNAi food from the MRC, [35] we also produced RNAi food specific for *hcp-1 *and *hcp-2*. Briefly, plasmid vectors for feeding RNAi were constructed by cloning either a 1.1 kb Spe I – Dra I fragment from the *hcp-1 *cDNA LM46-3 or a 1.6 kb Sal I fragment from the *hcp-2 *cDNA, yk19h10 into pPD129.36, which allows IPTG induction of double stranded RNA. Respective feeding vectors were transformed into the HTH115(BL23) *E. coli *strain and fed to animals, consistent with previously described RNAi methodology [35]. To conduct double RNAi experiments, both RNAi foods were plated, in equal amounts, onto the NGM IPTG plates. As a control we grew the *E. coli *strain, which has a plasmid with no insert, on identical NGM IPTG plates to account for any differences attributable to the vector. We conducted RT-PCR experiments to analyze the efficiency of RNAi by quantifying the level of *bub-3 *transcript in wild-type and *bub-3(RNAi) *animals. Methodology is as previously described [36]; we conducted at least three independent experiments and each experiment was performed in triplicate. Primer sequences used for RT-PCR reactions of *bub-3 *are as follow: 5'TGGGATCCTTTCAATCGGAAGC3' and 5'TTATTTCGGTCTGCTCCGGA3'. The P value was determined using the Mann-Whitney U Test.

### Spindle checkpoint assays

To assay whether specific genes are required for spindle checkpoint activity we exposed embryos to anoxia as previously described [21,29]. Briefly, the wild-type and *san-1(ok1580) *animals were grown, from the L1 larval stage to the 1 day old adult stage, on RNAi food (vector with no insert as a control or the specific strain to reduce expression of *bub-3*, *hcp-1*, or *hcp-2*). The adults were placed on a fresh RNAi plate and allowed to lay eggs for 1–2 hours. The adults were removed and the embryos were placed into anoxia, for 1 day, using the BioBag type A environmental chamber. The embryos were allowed to recover in air and assayed 24 hours later for the ability to hatch and three days later for the ability to reach adulthood. Four independent experiments with at least a total of 200 embryos were analyzed.

### DIC microscopy

The L1 larvae (N2, *san-1(ok1580)*, or *san-1(ok1580)*;*myo-2::GFP*) were grown to adulthood on appropriate RNAi plates (control, *bub-3*, or *mdf-2 *food). To analyze the developing pharynx in embryos, both the *san-1(ok1580);myo-2::GFP *and the *san-1(ok1580);hcp-1(RNAi);myo-2::GFP *adult animals were dissected and the embryos were placed onto 2.5% agarose pads for microscopy analysis. To analyze the morphology of control, *mdf-2(RNAi)*, *hcp-1(RNAi)*, *san-1(ok1580), san-1(ok1580);hcp-1(RNAi), san-1(ok1580);mdf-2(RNAi)*, and *san-1(ok1580);bub-3(RNAi) *animals, RNAi was conducted as stated above and the adult animals were placed on fresh RNAi plates and allowed to lay eggs for several hours. The embryos developed for three days at 20°C before DIC microscopy analysis. For all experiments the animals were placed on a 2.5% agarose pad and visualized using a motorized Zeiss Axioskop Fluorescent microscope and imaged using Openlab 3.17. At least three independent experiments were conducted.

### Indirect immunofluorescent microscopy

Wild-type and *san-1(ok1580) *animals were grown on appropriate RNAi food from L1 stage to adulthood. The adults were collected and dissected to release embryos. Embryos were collected, fixed and stained as previously described [22,29]. Briefly, the Phos H3 antibody recognizes the phosphorylated (Ser10) form of Histone H3 specific for mitotic cells (Upstate Biotechnology, Lake Placid NY)[30]; the mAb414 recognizes the nuclear pore complex (Babco, Berkeley, CA) [37]; mAb MPM-2 recognizes mitotic proteins located at the kinetochore, centrioles and P granules (DAKO, Carpinteria, CA) [32]; anti-HCP-3 detects HCP-3 [26]; and YL1/2 detects the spindle microtubules (Amersham Life Science, Little Chalfont, Buckinghamshire, England). Microscopy was done using either a Zeiss Axioskop or spinning disc confocal microscope (McBain, CA). Images were collected using the spinning disc confocal microscope and processed using Image J and Adobe Photoshop version 8.0.

### Live imaging to analyze mitosis

To analyze mitotic progression the chromosomes and centriole were used as markers, using the TH32 (*tbg-1::GFP, pie-1::GFP::H2B*) or PM115 (*san-1(ok1580)*;*tbg-1::GFP;pie-1::GFP::H2B) *strain. L1 larvae were grown on RNAi control or *hcp-1 *RNAi food to adulthood at 20°C. 1-day old gravid adults were dissected and embryos of the appropriate stage were collected by mouth pipet and placed on a 2.5% agarose pad. The temperature of the room in which the embryos were analyzed was at 21°C. Mitotic progression was quantified for 2-cell embryos using a spinning disc confocal microscope. To examine mitotic progression, the time it took, in seconds, to transition from the onset of prometaphase (as seen by nuclear envelope breakdown and condensed chromosomes) to the formation of the metaphase plate, and from the formation of the metaphase plate to the onset of anaphase was determined. Seven embryos were analyzed, using the Simple PCI Version 6 software program. The p-value (P) was determined using a standard student t-test. Movies, to demonstrate mitotic progression in the *san-1(ok1580)*;*tbg-1::GFP;pie-1::GFP::H2B *embryos, were obtained by analyzing live, young embryos using a spinning disc confocal microscope. Images were collected, processed using NIH Image, and imported into Quick Time for display.

## Competing interests

The author(s) declare that they have no competing interests.

## Authors' contributions

VAH conducted the indirect immunofluorescent assays and confocal analysis, produced and analyzed genetic crosses, assisted with experimental design and data interpretation, generated images, and edited the manuscript. AMS conducted genetic crosses, assisted with RNAi viability assays, conducted RT-PCR reactions and edited the manuscript. LLM conducted bioinformatics analysis, conceived experimental design, assisted with interpretation of data, provided necessary reagents for the study, generated images, and edited the manuscript. PAP conceived experimental design, conducted RNAi viability assays, analyzed phenotypes of mutants and RNAi animals, conducted genetic crosses, analyzed live cell images, generated images, and wrote the manuscript. All authors read and approved the final manuscript.

## Addendum

In the process of revision of this manuscript others also demonstrated the genetic interaction between the spindle checkpoint genes and *hcp-1 *in *C. elegans *[38].

## Supplementary Material

Additional file 1 The Y54G9A.6 gene encodes the putative BUB-3 protein. A multiple sequence alignment using ClustalW indicates that the Y54G9A.6 gene in *C. elegans *encodes a protein (BUB-3) with homology to the spindle checkpoint protein BUB3. The amino acid identity between the *C. elegans *putative BUB-3 protein and other BUB3 proteins is 44% for *H. sapiens*, 43% for *D. melanogastor *and 19% for *S. cerevisiae*. Identical amino acids have a black background shade. Click here for file

Additional file 2 Live cell imaging of *san-1(ok1580);hcp-1(RNAi);tbg-1::GFP;pie-1::GFP::H2B *embryos. A spinning disc confocal microscope to analyze chromosome segregation in the *san-1(ok1580);hcp-1(RNAi) *animals. Images were collected, processed using NIH Image and imported into Quick Time for display. In this embryo there are two blastomeres that progress through metaphase. One of the blastomeres displays normal mitotic progression (top metaphase blastomere) whereas another blastomere displays abnormal chromosome segregation (bottom metaphase blastomere) leading to anaphase bridging. Click here for file
